# Antibacterial Activity of 2-(2′,4′-Dibromophenoxy)-4,6-dibromophenol from *Dysidea granulosa*

**DOI:** 10.3390/md7030464

**Published:** 2009-09-22

**Authors:** Divya M. P. Shridhar, Girish B. Mahajan, Vijayendra P. Kamat, Chandrakant G. Naik, Rajashri R. Parab, Nidhi R. Thakur, Prabhu D. Mishra

**Affiliations:** 1 Bioorganic Chemistry, Chemical Oceanography Division, National Institute of Oceanography, Council of Scientific and Industrial Research (CSIR), Dona Paula, 403 004, Goa, India; E-Mail:cgnaik@nio.org (C.G.N.); 2 Dept. of Natural Products, Piramal Life Sciences Limited, Nirlon Complex, Goregaon, Mumbai, 400 063, India; E-Mails:girish.mahajan@piramal.com (G.B.M.);parabrajashri@rediffmail.com (R.R.P.);nidhithakur600@gmail.com (N.R.T.);prabhu.mishra@piramal.com (P.D.M.); 3 Dept. of Chemistry, Goa University, Taleigao, 403 206, Goa, India; E-Mail:vpkamat@unigoa.ac.in

**Keywords:** Dysidea granulosa, 2-(2′,4′-dibromophenoxy)-4,6-dibromophenol, antibacterial activity, methicillin resistant *Staphylococcus aureus*, vancomycin resistant *enterococci*

## Abstract

2-(2′,4′-Dibromophenoxy)-4,6-dibromophenol isolated from the marine sponge *Dysidea granulosa* (Bergquist) collected off the coast of Lakshadweep islands, Indian Ocean, exhibited potent and broad spectrum *in-vitro* antibacterial activity, especially against methicillin resistant *Staphylococcus aureus* (MRSA), methicillin sensitive *Staphylococcus aureus* (MSSA), vancomycin resistant *Enterococci* (VRE), vancomycin sensitive *Enterococci* (VSE) and *Bacillus* spp. Minimal inhibitory concentration (MIC) was evaluated against 57 clinical and standard strains of Gram positive and Gram negative bacteria. The observed MIC range was 0.117–2.5 μg/mL against all the Gram positive bacteria and 0.5–2 μg/mL against Gram negative bacteria. The *in-vitro* antibacterial activity observed was better than that of the standard antibiotic linezolid, a marketed anti-MRSA drug. The results establish 2-(2′,4′-dibromophenoxy)-4,6-dibromophenol, as a potential lead molecule for anti-MRSA and anti-VRE drug development.

## 1. Introduction

The striking rise in the prevalence of bacterial antibiotic resistance currently poses a serious threat to public health worldwide. Of particular concern are infections caused by methicillin-resistant *Staphylococcus aureus* (MRSA), penicillin-resistant *Streptococcus pneumoniae*, vancomycin-resistant *Enterococcus* [1] and *Mycobacterium tuberculosis* [2]. Many of these organisms have developed resistance to several classes of established antibiotics [1,2]. The most significant problem in clinical practice is the increase in incidence of MRSA infections. At present, the only effective treatment for multiple resistant MRSA infections is vancomycin. However, there are number of reports of emerging vancomycin resistance in some MRSA isolates [3]. Another group of clinically relevant multiple drug resistant bacteria that has emerged recently are *Enterococci*, some of which also exhibit vancomycin resistance. The appearance of vancomycin resistant *Enterococci* (VRE) infections has caused a dilemma upon physicians – linezolid and streptogramin combinations are the new drugs of choice for treating MRSA infections, but linezolid resistance have been reported in VRE and MRSA isolates [4]. Resistance to these oxazolidinone [5] streptogramin combinations [6,7] and various glycopeptides [5] requires expanded development of agents with alternative targets or modes of action. *Staphylococcus aureus* is the most clinically important of the Gram positive pathogens because of its exceptional virulence, stress tolerance, and capacity to accumulate antimicrobial resistances. MRSA is known as a major nosocomial pathogen which has also developed resistance to many other antibiotics. Moreover, MRSA and other *S. aureus* strains have been reported to acquire resistance to the last-resort antibiotic, vancomycin [8,9]. These facts suggest that *S. aureus* (MRSA) could fully acquire resistance to vancomycin in the near future.

The emergence and spread of resistant nosocomial and community-acquired pathogens is becoming a great menace to global public health. Therefore, a need is perceived to find new classes of antimicrobials. The search for new pharmacologically active agents obtained by screening natural sources has led to the discovery of many clinically useful drugs that play a major role in the treatment of human diseases. Approximately 60% of the antitumor and anti-infective agents that are commercially available or in late stages of clinical trials today are of natural product origin. Natural products are still major sources of innovative therapeutic agents for infectious diseases (both bacterial and fungal), cancer, lipid disorders and immunomodulation [10,11].

Given the diverse array of bioactive secondary metabolite chemical structures with a wide variety of biological activities isolated from marine sponges, natural product chemists have long been fascinated by these sessile marine invertebrates [11]. One of the most extensively studied species of sponge is the tropical marine sponge *Dysidea* sp. (family *Dysideidae*, Order *Dendroceratida*), a pinkish green sponge that occurs in a number of distinct chemotypes as a result of an association with symbiotic microorganisms. The chemistry reported from this sponge and related sponges in the genus, includes sesquiterpene spirolactol/lactones, tricyclic furans, some based on the furodysinin and furodysin skeletons and their oxidised derivatives, modified steroids, polychlorinated alkaloids, brominated diphenyl ethers and other metabolites. The polychlorinated alkaloids and brominated diphenyl ethers produced by the sponge have been shown to be associated with the filamentous cyanobacterium *Oscillatoria spongeliae*, and are therefore likely synthesized by the cyanobacterial symbionts [12–15].

Most halogenated phenols are found to be strongly antimicrobial. As a group, the halogenated bis(hydroxyphenyl) methanes and polybrominated diphenyl ethers exhibit a wide range of activities in bioassays, ranging from antibacterial activity (against *S. aureus* and *T. mentagrophytes*), to cytotoxicity (Ehrlich ascite tumor cells) [16,17]. Polybrominated biphenyl ethers from *Dysidea herbacea* are found to be active against eubacteria, as well as test strains of the unicellular marine cyanobacterium *Synechococcus* sp. [15]. Herein we have reported the isolation of 2-(2′,4′-dibromophenoxy)-4,6-dibromophenol from a new source *D. granulosa*, its potent *in-vitro* antibacterial activity, and as a possible lead molecule for drug development especially against strains of MRSA, methicillin sensitive *S. aureus* (MSSA), VRE and vancomycin sensitive *Enterococci* (VSE).

## 2. Results and Discussion

In the course of our programme of screening for new compounds from marine resources, 2-(2′,4′-dibromophenoxy)-4,6-dibromophenol was obtained from the sponge *Dysidea granulosa* as a colourless optically inactive crystalline solid, mp 179 °C. It showed a max. UV-Vis (MeOH) absorbance at 340 nm. IR (KBr) showed major peaks at 3,492.8 (phenolic O-H str), 3,084.0 (aromatic C-H str), 1,591.5 (aromatic C=C str), 1,463.9(C-H bend), 1,147.6, 1039.6 (C-O str), 918.1, 869.8, and 719.4 cm^−1^ (C-Br str). Spectral data including ^1^H-NMR (300 MHz, CDCl_3_), ^13^C-NMR, COSY, HMQC and HMBC of the compound is given in Table 1.

A quasimolecular [M-H] ^−^ ion peak was observed at m/z 500.7023 (calculated: 500.6982); the corresponding [2M-2H]^−^ peak was observed at m/z 1001.7274 (calculated: 1001.3964). The mass spectral fragmentation pattern with the respective spectral peaks at [M-H]^−^ at 496.7042, 498.7054, 500.7023, 502.7010 and 504.6974 (observed) and 496.7023, 498.7003, 500.6982, 502.6962 and 504.6942 (calculated) in the ratio 17.30:68.0:100.0:65.70:16.50 was indicative of the presence of four bromine atoms.

These spectral data were in good agreement with the spectral data reported in literature [15,18]. The two dimensional NMR data is reported here for the first time. 2-(2′,4′-Dibromophenoxy)-4,6-dibromophenol was observed to be a potent inhibitor of the growth of clinically relevant Gram positive bacteria, especially MRSA and VRE. Preliminary testing by the agar diffusion method revealed that the compound is strongly antibacterial, but has no inhibitory effect against fungal strains. To evaluate its *in-vitro* antibacterial potency and characterize its activity spectrum, the compound was tested against 57 bacterial test strains. The efficacy is measured in terms of minimum inhibitory concentration (MIC) and expressed in μg/mL.

The spectrum of bacterial test cultures includes 20 clinically derived and 10 standard strains of MRSA, two standard strains of MSSA; 11 clinical strains of VRE, four standard strains of VRE, five standard strains of VSE; one each standard strains of *E. coli, K. pneumoniae, S. typhi, S. flexneri* and *P. aeruginosa*, including three methicillin resistant strains of *S. aureus* 3066, E710, ATCC 33591 and two vancomycin resistant strains of *E. faecium* 02 D3 IP1, R-2 and one vancomycin resistant strain of *E. faecalis* ATCC 51299, which were subjected to antibacterial activity tests.

The compound showed potent, broad-spectrum *in-vitro* Gram-positive activity, as evident from Table 2. The compound exhibited no cross-resistance to other key antibiotics, which was evident from its potent activity against key antibiotic-resistant bacteria including MRSA, erythromycin-resistant *S. aureus*, and VRE (Table 3). *Escherichia coli* and *Pseudomonas* sp. were the least susceptible, with MIC values above 15 μg/mL (Table 2). However it was active against other Gram negative bacteria such as *Klebsiella pneumonia*, *Shigella flexneri* and *Salmonella typhi* (Table 2). The bromoether was tested by the agar diffusion assay against *C. albicans*, *A. fumigatus*, *C. tropicalis*, *C. glabrata* and was found to be inactive against fungi even up to 100 μg/mL. Thus, the compound specifically shows antibacterial activity. Our work has first time revealed the wide spectrum of activity of this compound. Its activity with variety of resistant strains confirmed that 2-(2′,4′-dibromophenoxy)-4,6-dibromophenol did not show cross resistance with widely marketed antibiotics such as erythromycin, β-lactams, vancomycin, teichoplanin, gentamicin and streptomycin. Thus it reflects that the type of bacterial strains used for antibacterial efficacy testing has no resistance to this compound. Therefore it could be a potential molecule to develop further to address the resistance issue.

## 3. Conclusions

This is the first report of 2-(2′,4′-dibromophenoxy)-4, 6-dibromophenol from the sponge *Dysidea granulosa* and the first attempt to exhaustively test the *in-vitro* activity of this molecule. Results obtained indicate that the compound shows good lead properties for antibacterial drug development and represent a promising agent for the treatment of *S. aureus* as well as enterococcal infections caused by drug-resistant strains.

## 4. Experimental Section

### 4.1. General Experimental Procedures

Column chromatography was carried out on silica gel 60–120 mesh, Sisco, India. Gel filtration on Sephadex LH20, Pharmacia Biotech, Sweden. Fractions were monitored on TLC kieselgel 60 F_254_ aluminum backed sheets and visualized under UV (254 nm) and iodine vapors. UV-VIS spectrum was recorded in methanol using a Shimadzu UV-2401PC spectrophotometer. IR spectrum was recorded on a Shimadzu FTIR-8201 PC spectrophotometer. NMR spectra (^1^H, ^13^C, COSY, HMQC and HMBC) were recorded on a Bruker Avance 300MHz instrument. Chemical shifts, relative to TMS as internal standard, are given in δ values. J values are given in Hz. Mass spectra were obtained on a QSTAR XL MS/MS system.

### 4.2. Collection of Animal Material and Chromatography

The sponge was collected off the coast of Lakshadweep Islands by scuba diving at a depth of about 8–10 meters and identified by Dr. P.A. Thomas of the Vizhingam Research Center of the Central Marine Fisheries Research Institute, Kerala, India. A voucher specimen is deposited at the National Institute of Oceanography, Dona Paula, Goa, India. The frozen sample of *D. granulosa* was lyophilized to obtain 200 g of dried sample, which was extracted successively with ethyl acetate and methanol (200 mL × 3 times each). These extracts were concentrated under reduced pressure and temperature to obtain the crude extracts. The ethyl acetate extract exhibited antimicrobial activity against pathogenic strains and was chromatographed over Sephadex LH20 (1:1 methanol-chloroform). Fractions that were similar in composition as shown by thin-layer chromatography were combined. The partially purified sample was further purified over silica gel (60–120 mesh) (2% ethyl acetate-petroleum ether) to afford the single major polybrominated secondary metabolite 2-(2′,4′-dibromophenoxy)- 4, 6-dibromophenol.

### 4.3. Antimicrobial Activity

Minimum Inhibitory Concentration (MIC) values were determined in Mueller Hinton Broth by the broth macrodilution method according to NCCLS guidelines, as per document no. M7-A5 [19]. The medium with a twofold serial dilution of compound in Mueller Hinton broth was inoculated with 10^5^ colony forming units/mL of test culture and incubated at 37 °C for 24 hrs. The MIC of the bromoether was determined against 57 test organisms including different clinical and standard strains of *S. aureus*, both MRSA and MSSA, clinical and inhouse strains of *Enterococci*, both VRE and VSE and some gram negative strains. American type culture collection (ATCC) strains were also screened. Linezolid (Glenmark Pharmaceuticals Ltd, India) was used as control agent.

## Figures and Tables

**Figure1:**
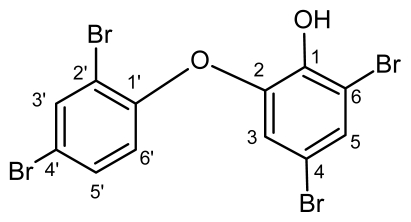
Structure of 2-(2′,4′-dibromophenoxy)-4,6-dibromophenol.

**Table 1:** ^1^H-, ^13^C-NMR, COSY and HMBC of 2-(2′,4′-dibromophenoxy)-4,6-dibromophenol.

Carbon No.	^13^C-NMR δ_C_, ppm	^1^H-NMR δ_H_, ppm	COSY correlations	HMBC correlations

1	138.6	-		
2	150.3	-		
3	119.7	7.20 (d, 2.3Hz)		1, 2, 5
4	117.3	-		
5	127.8	7.34 (d, 2.3Hz)		4, 6
6	119.8	-		
1′	152.0	-		
2′	112.6	-		
3′	136.2	7.77 (d, 2.3Hz)		1′, 5′
4′	116.0	-		
5′	131.5	7.29 (dd, 8.8Hz)	H6′	3′, 1′
6′	115.9	6.43 (d, 8.8Hz)	H5′	1′, 2′

**Table 2:** Antimicrobial activity of 2-(2′,4′-dibromophenoxy)-4,6-dibromophenol.

Test Organisms	MIC Range (μg/mL)	MIC_50_ (μg/mL)	MIC_90_ (μg/mL)

S. aureus* MRSA (20)	0.313–1.25	0.313	0.625
S. aureus** MRSA (10)	0.313–10	0.313	0.625
S. aureus** MSSA (2)	0.117–0.313	-	-
Enterococci* VRE (11)	0.625–2.5	0.625	1.25
E. faecalis** VSE (4)	1.25–2.5	-	-
E. faecium** VSE (1)	1.25	-	-
E. faecium** VRE (3)	1.25	-	-
E. faecalis** VRE (1)	1.25	-	-
E. coli** (1)	>15	-	-
K. pneumoniae** (1)	2	-	-
Salmonella typhi** (1)	0.5	-	-
*Shigella flexneri* Type IV** (1)	2	-	-
P. aeruginosa** M-35 (1)	>15	-	-

* Clinical derived strains,

** Standard strains procured from culture banks. -: Not applicable.

# The numbers in brackets indicate the no. of strains tested.

**Table 3:** Antimicrobial activity of 2-(2′,4′-dibromophenoxy)-4,6-dibromophenol and linezolid towards resistant strains.

Test organisms	MIC (μg/mL)	
	Compound	Linezolid

*S. aureus* (3066), MRSA, Ery^R^	0.313	0.313
*S. aureus* (E710), MRSA, Ery^R^	0.625	2.5
*S. aureus* ATCC 33591, MRSA	0.313	2.5
*E. faecium* (02 D3 IP1), Vanco^R^, Teicho^R^	1.25	2.5
*E. faecium* (R-2), Vanco^R^ (VanA)	1.25	2.5
*E. faecalis* ATCC 51299, VRE, Gen^R^, Strept^R^ (VanB)	2.5	2.5
